# A Taxonomic Appraisal of Bambusicolous Fungi in Occultibambusaceae (Pleosporales, Dothideomycetes) with New Collections from Yunnan Province, China

**DOI:** 10.3390/life11090932

**Published:** 2021-09-07

**Authors:** Hong-Bo Jiang, Rungtiwa Phookamsak, Kevin D. Hyde, Peter E. Mortimer, Jian-Chu Xu, Pattana Kakumyan, Samantha C. Karunarathna, Jaturong Kumla

**Affiliations:** 1 Department of Biology, Faculty of Science, Chiang Mai University, Chiang Mai 50200, Thailand; hongbo-j@hotmail.com; 2 Centre for Mountain Futures (CMF), Kunming Institute of Botany, Kunming 650201, China; jomjam.rp2@gmail.com (R.P.); peter@mail.kib.ac.cn (P.E.M.); jxu@mail.kib.ac.cn (J.-C.X.); 3 Center of Excellence in Fungal Research, Mae Fah Luang University, Chiang Rai 57100, Thailand; kdhyde3@gmail.com; 4 School of Science, Mae Fah Luang University, Chiang Rai 57100, Thailand; pattana.kak@mfu.ac.th; 5 CIFOR-ICRAF China Program, World Agroforestry (ICRAF), Kunming 650201, China; 6 Honghe Center for Mountain Futures, Kunming Institute of Botany, Chinese Academy of Sciences, Honghe County 654400, China; 7 Yunnan Key Laboratory for Fungal Diversity and Green Development, Kunming Institute of Botany, Chinese Academy of Sciences, Kunming 650201, China; 8 Research Center of Microbial Diversity and Sustainable Utilization, Faculty of Science, Chiang Mai University, Chiang Mai 50200, Thailand; 9 Innovative Institute for Plant Health, Zhongkai University of Agriculture and Engineering, Guangzhou 510000, China

**Keywords:** *Occultibambusa*, one new record, *Seriascoma*, taxonomy, two new taxa

## Abstract

During our ongoing studies of bambusicolous fungi in southwest China and Thailand, three saprobic pleosporalean taxa were discovered on bamboos in Yunnan Province of China. *Occultibambusa hongheensis* and *Seriascoma bambusae* spp. nov. are introduced based on morphological characteristics coupled with multi-locus phylogenetic analyses of combined LSU, SSU, TEF1-α, RPB2 and ITS sequence data. *Occultibambusa kunmingensis* is also reported from a terrestrial habitat for the first time. Comprehensive descriptions, color photo plates of micromorphology, and a phylogenetic tree showing the placements of these three taxa are provided. In addition, synopsis tables of *Occultibambusa* and *Seriascoma* with morphological features are also provided.

## 1. Introduction

Occultibambusaceae is a well-resolved family with a strong morpho-molecular basis. The family accommodates five genera viz. *Brunneofusispora* S.K. Huang & K.D. Hyde, *Neooccultibambusa* Doilom & K.D. Hyde, *Occultibambusa* D.Q. Dai & K.D. Hyde, *Seriascoma* Phookamsak, D.Q. Dai & K.D. Hyde, and *Versicolorisporium* Sat. Hatak., Kaz. Tanaka & Y. Harada [1,2,3]. Occultibambusaceae was introduced by Dai et al. [4] to accommodate *Neooccultibambusa*, *Occultibambusa*, *Seriascoma*, and *Versicolorisporium*, with *Occultibambusa* as the type genus. *Brunneofusispora* became a new member of Occultibambusaceae [5]. As a result of thriving molecular techniques, all genera in Occultibambusaceae have been resolved using multi-gene phylogeny [4,5,6,7].

Occultibambusaceae is characterized by solitary, immersed, subglobose to conical, greyish to dark brown, uni- or multi-loculate ascostromata, scattered or in small groups, with papillate, or protruding ostioles, bitunicate, fissitunicate, (6)–8-spored, cylindrical to clavate asci with short, furcate or bulb-like pedicels, and 1–3-seriate, fusiform, hyaline, or pale brown to dark brown, 1–3-septate ascospores with or without a sheath [1,4,5,6,7,8,9,10,11,12,13]. *Brunneofusispora*, *Occultibambusa*, *Seriascoma*, and *Versicolorisporium* were reported to have coelomycetous asexual morphs [4,14,15,16], while *Neooccultibambusa* forms chlamydospores in culture or has as hyphomycetous asexual morphs [6,9,17].

Occultibambusaceae is a small family with 18 species [18]. To date, this family has been reported from China, Italy, Japan, and Thailand [4,5,6,7,8,9,10,11,12,13,14,15,16,17,19]. With the exception of species of *Neooccultibambusa* and *Brunneofusispora*, most species of Occultibambusaceae are saprobes on dead bamboo [4,8,10,12,13]. *Neooccultibambusa* has been found on a wide variety of hosts such as *Ammophila* sp., *Pandanus* sp. and *Tectona grandis* [6,9,11,17]. *Brunneofusispora* was reported on dead wood and *Clematis* sp. in terrestrial habitats and decaying wood submerged in freshwater habitats [5,7,15,16].

*Occultibambusa* is typified by *O. bambusae* D.Q. Dai & K.D. Hyde and is characterized by solitary or gregarious, raised, immersed, subglobose to conical, dark brown, uni-loculate, coriaceous ascostromata with black, papillate ostioles, bitunicate, fissitunicate, eight-spored, broadly cylindrical to clavate asci, and fusiform, pale brown to brown, 1–(3)-septate ascospores, with or without a sheath [4,8,10,12,13]. Eight species are accommodated in *Occultibambusa* [18]. However, only *O. fusispora* Phookamsak, D.Q. Dai & K.D. Hyde has a known coelomycetous asexual morph and is characterized by multi-loculate, eustromatic, immersed, solitary to gregarious, globose to subglobose, black conidiomata with long papillate necks and enteroblastic, phialidic, determinate, cylindrical to ampulliform, hyaline, smooth, aseptate conidiogenous cells bearing oblong to cylindrical, hyaline, aseptate, guttulate, smooth-walled conidia [4].

*Seriascoma* is typified by *S. didymosporum* Phookamsak, D.Q. Dai, S.C. Karunarathana & K.D. Hyde. *Seriascoma didymosporum* and *S. yunnanense* Rathnayaka & K.D. Hyde are accommodated in the genus [1,12,18]. *Seriascoma* is characterized by solitary or gregarious, erumpent, subglobose or elongated, uni- to multi-loculate, coriaceous ascostromata, immersed under a clypeus, bitunicate, fissitunicate, eight-spored, clavate asci with short to long furcate pedicels and 1–3-seriate, fusiform, asymmetric, 1-septate, hyaline ascospores with or without a sheath [4,12,13]. The asexual morph of this genus has only been reported in *S. didymosporum* and is characterized by eustromatic, solitary to gregarious, semi-immersed to erumpent, conical, black, uni- to multi-loculate conidiomata and enteroblastic, phialidic, determinate, cylindrical to ampulliform, hyaline, aseptate, smooth-walled conidiogenous cells bearing oblong, hyaline, aseptate, smooth-walled conidia [4].

During our studies on bambusicolous fungi in southwest China and Thailand, three new fungal strains belonging to Occultibambusaceae were collected and isolated from Yunnan Province in China. This study introduces two novel species in *Occultibambusa* and *Seriascoma* based on multi-locus phylogenetic analyses and morphological characteristics. In addition, *Occultibambusa kunmingensis* C.X. Liu, H. Zhang & K.D. Hyde is reported from a terrestrial environment for the first time.

## 2. Materials and Methods

### 2.1. Collection, Examination, Isolation and Preservation

Dead bamboo branches and culms were collected from Mengla County, Xishuangbanna Dai Autonomous Prefecture, Yunnan Province, China in January 2019 and Honghe County, Honghe Hani and Yi Autonomous Prefecture, Yunnan Province, China, in October 2020. Samples were stored in plastic Ziploc bags and taken to the laboratory at Kunming Institute of Botany, CAS, Kunming, Yunnan Province, China for observation and examination following the method described by Senanayake et al. [20]. Fungal fruiting bodies on host substrates were visualized under a Motic SMZ 140 series dissecting stereoscope and photographed by digital camera. Vertical sections of ascostromata and conidiomata and other micro-morphological characteristics (e.g., peridium, pseudoparaphyses, asci, ascospores, conidiogenous cells and conidia) were observed and captured with a Nikon ECLIPSE Ni compound microscope connected with a Canon EOS 600D digital camera. The Tarosoft (R) Image FrameWork version 0.9.7 program was used to measure the size (10–20 measurements of each structure) of fungal characteristics. Adobe Photoshop CS6 software (Adobe Systems Inc., San Jose, CA, USA) was used to edit and combine photographic plates. Ex-type living culture of *Occultibambusa fusispora* (MFLUCC 11-0127) was also loaned from Mae Fah Luang University Culture Collection, Chiang Rai, Thailand (MFLUCC). It was aseptically sub-cultured in a laminar flow and incubated at room temperature (20–25 °C) for sequencing. Specimens of new taxa and new collections obtained for this study have been deposited in the herbarium of Cryptogams Kunming Institute of Botany Academia Sinica (KUN-HKAS), Yunnan, China and the Herbarium Mycologicum Academiae Sinicae (HMAS), Beijing, China. Living cultures have been deposited in the China General Microbiological Culture Collection Center, Beijing, China (CGMCC) and Kunming Institute of Botany Culture Collection, Kunming, China (KUMCC). Facesoffungi and Index Fungorum numbers have been registered for the newly described taxa [21,22]. New species have been established based on the guidelines of Jeewon and Hyde [23].

### 2.2. DNA Extraction, PCR Amplification and Sequencing

Genomic DNA of new fungal isolates and a loaned strain of *Occultibambusa fusispora* (MFLUCC 11-0127) was extracted from fresh mycelia using Biospin Fungus Genomic DNA extraction kit (BioFlux^®^, Hangzhou, China) following the manufacturer’s instructions. DNA amplification was performed by polymerase chain reaction (PCR). Five primer pairs viz. ITS5/ITS4 [24], LR0R/LR5 [25], NS1/NS4 [24], EF1-983F/EF1-2218R [26] and fRPB2-5F/fRPB2-7cR [27] were used to amplify the fragments of the internal transcribed spacers (ITS1-5.8S-ITS2), the 28S large subunit rDNA (LSU), the 18S small subunit rDNA (SSU), the translation elongation factor 1-alpha (TEF1-α), and the partial RNA polymerase second largest subunit (RPB2), respectively. PCR was carried out based on 25 µL total volume per reaction, containing 2 µL of fungal genomic DNA, 1 µL of each forward and reverse primer, 12.5 µL of 2 × Power Taq PCR Master Mix (a mixture of EasyTaqTM DNA Polymerase, dNTPs, and optimized buffer; Beijing BioTeke Corporation, China) and 8.5 µL of sterilized double-distilled water (ddH_2_O). The PCR thermal cycle profiles for ITS, LSU, SSU, and TEF1-α gene was processed under the following conditions: an initial denaturation at 94 °C for 3 min, followed by 40 cycles of denaturation at 94 °C for 30 s, annealing at 55 °C for 50 s, elongation at 72 °C for 1 min, and a final extension at 72 °C for 10 min, and finally kept at 4 °C. We followed the PCR thermal cycle profiles for RPB2 gene in Jiang et al. [28]. Final PCR products were sent to TsingKe Biological Technology (Beijing) Co., Ltd., China for PCR purification and sequencing. The Sanger dideoxy sequencing method was used for the new strains. The quality of sequences was checked by both manual and FinchTV v. 1.4.0 (http://www.geospiza.com/Products/finchtv.shtml (accessed on 5 April 2021)).

### 2.3. Alignment and Phylogenetic Analyses

The nucleotide BLAST search (https://blast.ncbi.nlm.nih.gov/Blast.cgi (accessed on 10 April 2021)) was applied to discover taxa closely related to our three new isolates (KUMCC 21-0019, KUMCC 21-0020 and KUMCC 21-0021). Similarity indices from the BLAST search indicated that KUMCC 21-0019 and KUMCC 21-0020 belong to *Occultibambusa* (Occultibambusaceae) and KUMCC 21-0021 belongs to *Seriascoma* (Occultibambusaceae). Therefore, to reveal accurate phylogenetic placements of our three new isolates, multi-gene phylogeny of Occultibambusaceae and the closely related family Nigrogranaceae (Pleosporales, Dothideomycetes) were done based on maximum-likelihood and Bayesian inference methods. DNA sequences of representative taxa in Occultibambusaceae and Nigrogranaceae are shown in Table 1. Sequence alignments and phylogenetic analyses were carried out following methods described by Dissanayake et al. [29]. Preliminarily individual DNA sequence matrixes were aligned via the online platform, MAFFT v. 7.475 [30]. Aligned sequence datasets were trimmed by TrimAl v. 1.3 via the web server phylemon 2 (http://phylemon.bioinfo.cipf.es/utilities.html (accessed on 20 April 2021)) and then improved where necessary using BioEdit v. 6.0.7 [31], i.e., complementing the missing bases at the start and end of the consensus sequence. Individual gene datasets were analyzed by maximum likelihood criteria in order to compare the congruence of tree topologies.

Maximum-likelihood (ML) analysis was performed via the online portal CIPRES Science Gateway v. 3.3 [32], with RAxML-HPC v.8 on XSEDE (8.2.12) tool, using default settings but following the adjustments: the GAMMA nucleotide substitution model and 1000 rapid bootstrap replicates. The evolutionary model of nucleotide substitution for Bayesian inference (BI) analysis was selected independently for each locus using MrModeltest 2.3 [33]. GTR+I+G was the best-fit for LSU, TEF1-α, and RPB2 loci under the Akaike Information Criterion (AIC), while the GTR+G substitution model was the best-fit for the ITS locus and HKY+I+G was the best-fit for the SSU locus. BI analysis was performed via MrBayes v. 3.1.2 [34]. Markov chain Monte Carlo sampling (MCMC) was used to determine posterior probabilities (PP) [35,36]. Six simultaneous Markov chains were run for 1,000,000 generations and trees were sampled every 100th generation. The 0.15 “temperature” value was set in MCMC heated chain. All sampled topologies beneath the asymptote (20%) were discarded as part of a burn-in procedure and the remaining 8000 trees were used for calculating posterior probabilities (PP) in the 50% majority rule consensus tree (when split frequency lower than 0.01).

The tree topologies generated in this study were visualized on FigTree v. 1.4.0 (http://tree.bio.ed.ac.uk/software/figtree/ (accessed on 20 April 2021)). The phylogram was edited and redrawn by using Microsoft Office PowerPoint 2016 (Microsoft Inc., Redmond, WA, USA) and converted to tiff file on Adobe Photoshop CS6 software (Adobe Systems Inc., San Jose, CA, USA). New sequences generated from the present study were deposited in GenBank (Table 1). The final alignment and phylogram were submitted to TreeBASE (submission ID: 28553, https://www.treebase.org/ (accessed on 20 July 2021)).

**Table 1:** Taxa names, strain numbers, and GenBank accession numbers of taxa used for the present phylogenetic analyses.

Taxa Names	Strain Numbers	Origin	Substrate/Host	GenBank Accession Numbers					Refs.
				LSU	SSU	TEF1-α	RPB2	ITS	
*Brunneofusispora clematidis*	**MFLUCC 17-2070**	Chiang Rai, Thailand	Dead stems of *Clematis subumbellata*	MT214570	NG_070658	MT394629	MT394692	MT310615	[7]
*Brunneofusispora hyalina*	**MFLUCC 21-0008**	Chiang Mai, Thailand	Decaying wood	MW287234	MW485613	MW512606	MW512609	MW260330	[16]
*Brunneofusispora sinensis*	**KUMCC 17-0030**	Yunnan, China	Dead wood	MH393557	MH393556	MH395329	/	MH393558	[5]
*Brunneofusispora sinensis*	MFLUCC 20-0016	Yunnan, China	Dead branches of *Magnolia denudata*	MT159624	MT159636	MT159607	MT159613	MT159630	[15]
*Brunneofusispora* sp.	X135	China	*Ageratina adenophora*	/	/	/	/	MK304223	[16]
*Massarina rubi*	CBS 691.95	Austria	*Ulmus glabra*	FJ795453	GU456301	/	FJ795470	/	Unknown
*Massarina rubi*	MUT 4323	Italy	Rhizomes of *Posidonia oceanica*	KF636772	/	/	/	KF636766	Unpublished
*Massarina rubi*	MUT 4887	Italy	*Flabellia petiolata*	KP671721	KT587318	/	/	KR014359	Unpublished
*Massarina* sp.	MUT 4860	Italy	*Flabellia petiolata*	KP671730	KT587325	/	/	KR014362	Unpublished
*Neooccultibambusa chiangraiensis*	**MFLUCC 12-0559**	Chiang Rai, Thailand	Dead twigs of *Tectona grandis*	KU764699	NG_061230	KU872761	/	NR_154238	[6]
*Neooccultibambusa jonesii*	**MFLUCC 16-0643**	Italy	Dead and stems of *Ammophila arenaria*	NG_059741	NG_062422	/	/	/	[9]
*Neooccultibambusa pandanicola*	**KUMCC 17-0179**	Yunnan, China	Dead leaves of *Pandanus utilis*	MG298940	MG298942	MG298943	MG298944	MG298941	[17]
*Neooccultibambusa thailandensis*	**MFLUCC 16-0274**	Prachuap Khiri Khan, Thailand	Dead leaf of *Pandanus* sp.	MH260308	MH260348	MH412780	MH412758	MH275074	[11]
*Nigrograna mackinnonii*	E5202H	Ecuador	Dead stems of *Guazuma ulmifolia*	KJ605422	JX264155	JX264154	JX264156	JX264157	Unpublished
*Nigrograna obliqua*	MRP	Austria	*Ribes uva-crispa*	KX650561	/	KX650532	KX650581	KX650561	[37]
*Nigrograna obliqua*	BW4	Austria	A twig of *Sambucus racemosa*	KX650557	/	KX650529	/	KX650557	[37]
*Occultibambusa aquatica*	**MFLUCC 11-0006**	Chiang Rai, Thailand	Bamboo	KX698110	KX698112	/	/	/	[8]
*Occultibambusa bambusae*	MFLUCC 11-0394	Chiang Mai, Thailand	Dead culms of bamboo	KU863113	KU872117	KU940194	KU940171	KU940124	[4]
*Occultibambusa bambusae*	**MFLUCC 13-0855**	Chiang Rai, Thailand	Dead culms of bamboo	KU863112	KU872116	KU940193	KU940170	KU940123	[4]
*Occultibambusa chiangraiensis*	**MFLUCC 16-0380**	Chiang Rai, Thailand	Dead stems of *Bambusoideae* sp.	KX655546	NG_062421	KX655561	KX655566	/	[8]
*Occultibambusa fusispora*	**MFLUCC 11-0127**	Chiang Rai, Thailand	Dead branches of bamboo	NG_059669	/	KU940195	KU940172	NR_154340	[4]
*Occultibambusa fusispora*	**MFLUCC 11-0127II**	Chiang Rai, Thailand	Dead branches of bamboo	MZ329032	MZ329028	MZ325466	MZ325469	MZ329036	This study
*Occultibambusa hongheensis*	**KUMCC 21-0020**	Yunnan, China	Dead branches of bamboo	MZ329033	MZ329029	MZ325467	/	MZ329037	This study
*Occultibambusa jonesii*	**GZCC 16-0117**	Guizhou, China	Dead culms of bamboo	NG_066381	NG_065104	KY814756	KY814758	/	[10]
*Occultibambusa kunmingensis*	**KUN-HKAS 102151**	Yunnan, China	Decaying bam	MN913733	MT864342	MT954407	MT878453	MT627716	[13]
*Occultibambusa kunmingensis*	KUMCC 21-0019	Yunnan, China	Submerged bamboo	MZ329034	MZ329030	/	/	MZ329038	This study
*Occultibambusa maolanensis*	**GZCC 16-0116**	Guizhou, China	Dead culms of bamboo	KY628323	KY628325	KY814757	KY814759	/	[10]
*Occultibambusa pustula*	**MFLUCC 11-0502**	Chiang Rai, Thailand	Dead culm of bamboo	KU863115	NG_062419	/	/	NR_154341	[4]
*Ohleria modesta*	MGC	Spain	Branches of *Chamaecytisus proliferus*	KX650562	/	KX650533	KX650582	KX650562	[37]
*Ohleria modesta*	**OM**	Spain	Branches of *Chamaecytisus proliferus*	KX650563	KX650513	KX650534	KX650583	KX650563	[37]
*Seriascoma bambusae*	**KUMCC 21-0021**	Yunnan, China	Dead culms of bamboo	MZ329035	MZ329031	MZ325468	MZ325470	MZ329039	This study
*Seriascoma didymosporum*	**MFLUCC 11-0179**	Chiang Rai, Thailand	Dead culms of bamboo	NG_059670	KU872119	KU940196	KU940173	NR_154433	[4]
*Seriascoma didymosporum*	MFLUCC 11-0194	Chiang Rai, Thailand	Dead culms of bamboo	KU863117	KU872120	KU940197	KU940174	KU940128	[4]
*Seriascoma* sp.	KUMCC 21-0007	Yunnan, China	Dead branches of bamboo	MW981347	MZ325471	MZ325472	MZ325473	MW981351	[38]
*Seriascoma yunnanense*	**MFLU 19-0690**	Yunnan, China	Dead branches of bamboo	NG_068303	MN174694	MN381858	MN210324	/	[12]
*Versicolorisporium triseptatum*	**HHUF 28815**	Honshu, Japan	Dead culms of *Pleioblastus chino*	NG_042318	NG_060995	/	/	NR_119392	[14]

The ex-type strains are in bold. **Abbreviations: GZCC:** Guizhou Culture Collection, Guizhou, China; **HHUF:** Herbarium of Hirosaki University, Japan; **KUMCC:** Kunming Institute of Botany Culture Collection, Kunming, China; **KUN**-**HKAS**: Herbarium of Cryptogams Kunming Institute of Botany Academia Sinica, Yunnan, China; **MFLU**: Herbarium of Mae Fah Luang University, Chiang Rai, Thailand; **MFLUCC**: Mae Fah Luang University Culture Collection, Chiang Rai, Thailand; **MUT**: Mycotheca Universitatis Taurinensis, Torino, Italy.

## 3. Results

### 3.1. Phylogenetic Analyses

**Figure 1:**
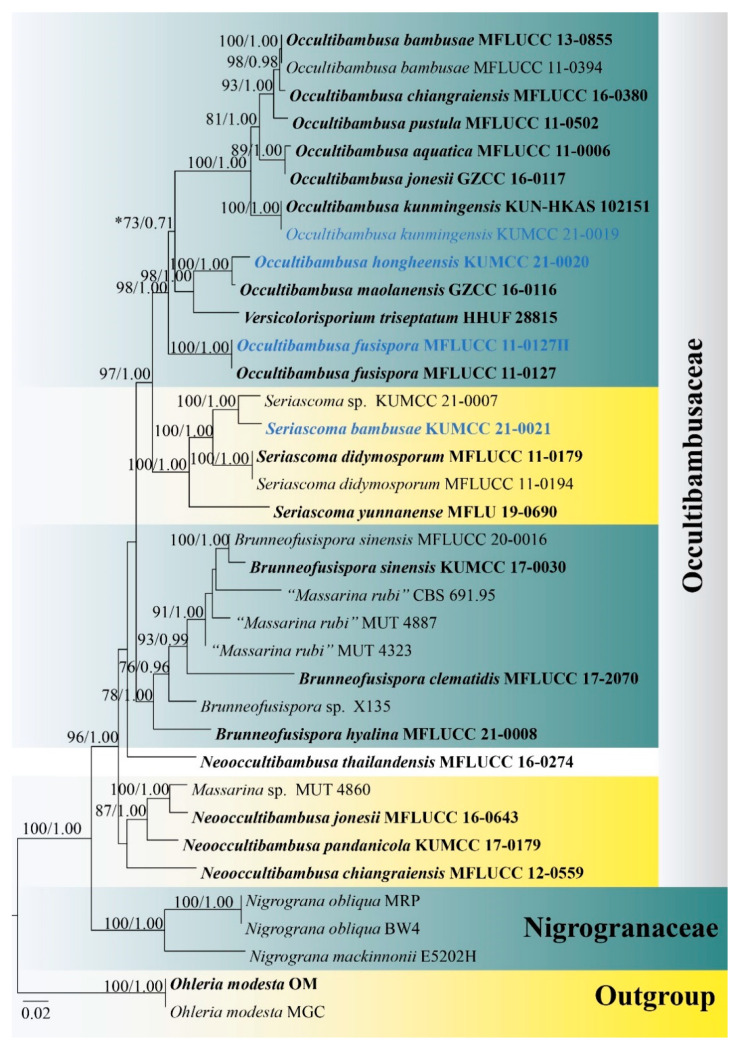
RAxML tree based on LSU, SSU, TEF1-α, RPB2, and ITS sequence matrix representing the phylogenetic relationships of taxa in Occultibambusaceae. The tree is rooted to *Ohleria modesta* (MGC and OM). Bootstrap support values for ML equal to or greater than 70% and the Bayesian posterior probabilities equal to or higher than 0.95 PP are indicated above the nodes as ML/PP. Ex-type strains are in bold and the new species and new record are indicated in blue. * These values 73/0.71 are indicated on the node to discuss the separation of *Occultibambusa* taxa.

The combined LSU, SSU, TEF1-α, RPB2 and ITS sequence matrix comprises 36 strains of representative species in Occultibambusaceae, the closely related family Nigrogranaceae, and *Ohleria modesta* (MGC and OM) as the outgroup. The dataset consists of 4308 total characters, including gaps (LSU: 1–832 bp, SSU: 833–1855 bp, TEF1-α: 1856–2791 bp, RPB2: 2792–3855 bp, ITS: 3856–4308 bp). The best scoring ML tree was selected to represent the phylogenetic relationships of two new taxa and a new record taxon with other representative taxa in Occultibambusaceae (Figure 1), with the final ML optimization likelihood value of −20,955.880345 (ln). All free model parameters were estimated by GTRGAMMA model, with 1331 distinct alignment patterns and 26.65% undetermined characters or gaps. Estimated base frequencies were as follows: A = 0.246035, C = 0.250866, G = 0.268453, T = 0.234646, with substitution rates AC = 2.237705, AG = 4.680757, AT = 1.669097, CG = 1.580519, CT = 10.758320, GT = 1.000000. The gamma distribution shape parameter alpha = 0.169891 and the Tree-Length = 1.401029. The final average standard deviation of split frequencies at the end of total MCMC generations was calculated as 0.003559 in BI analysis.

Tree topologies generated based on ML and BI analyses were similar in the present study and the ML phylogenetic tree is shown in Figure 1. All genera in Occultibambusaceae formed well-resolved clades, except for *Versicolorisporium*, which clustered within *Occultibambusa*. *Neooccultibambusa thailandensis* formed an independent lineage separated from other *Neooccultibambusa* species. Multi-locus phylogeny demonstrated that the new isolates (KUMCC 21-0019, KUMCC 21-0020 and KUMCC 21-0021) belong to Occultibambusaceae. KUMCC 21-0019 and KUMCC 21-0020 clustered within the *Occultibambusa* clade, and KUMCC 21-0021 grouped with the other *Seriascoma* species. KUMCC 21-0020 is sister to *O. maolanensis* with high statistical support (100% ML, 1.00 PP). Thus, *Occultibambusa hongheensis* sp. nov. (KUMCC 21-0020) is hereby introduced. The strain KUMCC 21-0019 shared the same branch length with the type strain of *O. kunmingensis* (KUN-HKAS 102151) with high statistical support (100% ML, 1.00 PP). Therefore, the new strain KUMCC 21-0019 is identified as *O. kunmingensis*, whereas *O. fusispora* (MFLUCC 11-0127II) was re-sequenced from the ex-type living culture and the newly generated sequences were found to be consistent with *O. fusispora* (MFLUCC 11-0127), clarifying the correctness of phylogenetic placement of *O. fusispora* as basal to *Occultibambusa*. Strain KUMCC 21-0021 formed a distinct subclade with *Seriascoma* sp. (KUMCC 21-0007) with high statistical support (100% ML, 1.00 PP). Hence, *Seriascoma bambusae* (KUMCC 21-0021) is introduced as a new species.

### 3.2. Taxonomy

#### 3.2.1. *Occultibambusa hongheensis* H.B. Jiang, K.D. Hyde & Phookamsak, sp. nov.

**Figure 2:**
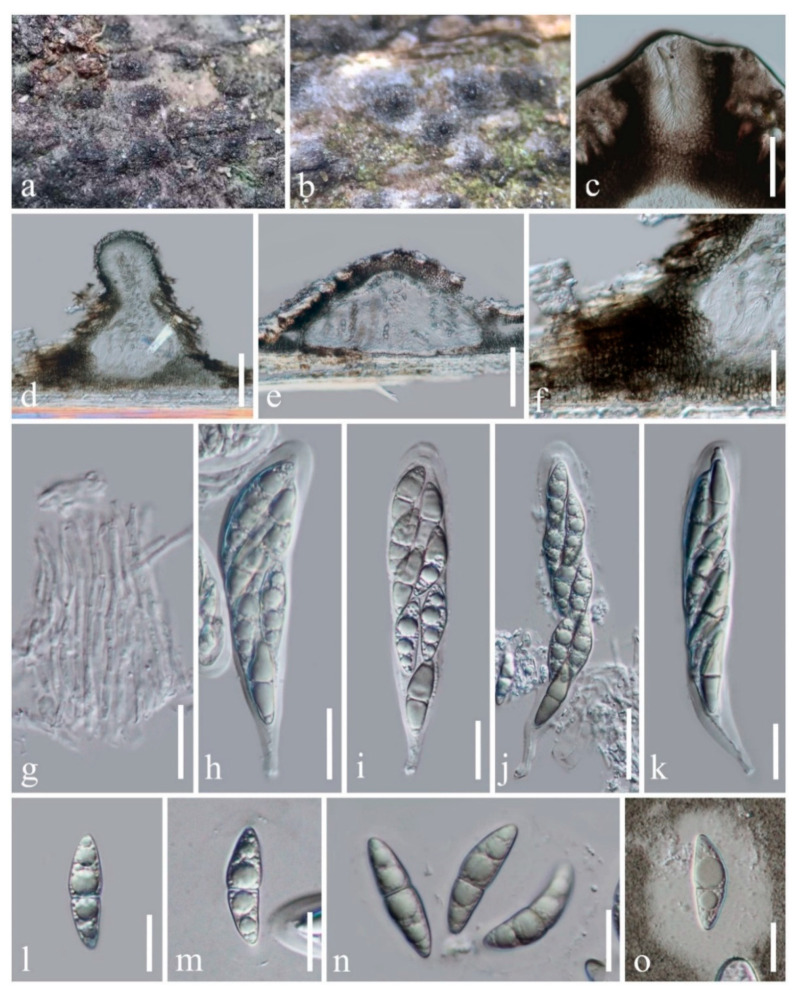
*Occultibambusa hongheensis* (HMAS 249944, holotype). (**a**,**b**) Appearance of ascostromata on the host; (**c**) Periphysate ostiole; (**d**,**e**) Vertical sections of ascostroma; (**f**) Peridium arranged in *textura angularis*, with palisade-like cells at the side; (**g**) Pseudoparaphyses; (**h**–**k**) Asci; (**l**–**o**) Ascospores [(**o**) Ascospore with mucilaginous sheath stained by Indian ink]. Scale bars: (**d**,**e**) = 100 μm; (**c**,**f**) = 50 μm; (**j**) = 30 μm; (**g**–**i**,**k**) = 20 μm; (**l**–**o**) = 15 μm.

Index Fungorum number: IF558429; Facesoffungi number: FoF 09884; Figure 2

Etymology: The specific epithet “*hongheensis*” refers to the location, Honghe, Yunnan Province of China, where the new species was collected.

Holotype: HMAS 249944

*Saprobic* on dead branches of bamboo. Sexual morph: *Ascostromata* 180–340 μm high, 400–550 μm diam., solitary or gregarious, immersed under host cortex, ampulliform, conical to subglobose, flattened at the base, uni- to bi-loculate, black, coriaceous, with 80–125 μm broad, central, periphysate ostiole. *Peridium* 40–130 μm thick, of unequal thickness, thin at the base, thick at sides, composed of several layers of pseudoparenchymatous cells of *textura angularis*, with palisade-like cells on the sides, outer layers consisting of dark brown pseudoparenchymatous cells, fused with host tissues, paler towards the inner layers. *Hamathecium* dense, composed of 1–2 μm wide, septate, branched, anastomosed, cellular pseudoparaphyses. *Asci* (78–)80–130(–137) × (18–)19–23(–25) μm (*x* = 107.5 × 21.5 μm, *n* = 20), eight-spored, bitunicate, fissitunicate, cylindric-clavate to clavate, with a short pedicel, apically rounded with a distinct ocular chamber. *Ascospores* (25–)27–30 × (5.5–)8–9(–10) μm (*x* = 28.8 × 8.4 μm, *n* = 20), partially overlapping 2-seriate, fusiform, 1-septate, slightly constricted at the septum, asymmetrical, upper cell broader and longer than the lower cell, straight to somewhat curved, hyaline when young and becoming pale brown when mature, smooth-walled, with guttules, surrounded by a broad mucilaginous sheath. Asexual morph: Undetermined.

Culture characteristics: Ascospores germinating on PDA within 24 h and germ tubes produced from both cells of ascospores. Colonies were grown on PDA, reaching 30 mm after four weeks at room temperature (10–20 °C), under normal light conditions, colonies on PDA cottony, circular, raised, dense, pale grey to dark grey from above and below. Mycelium superficial to immersed in media, with branched, septate, smooth hyphae.

Material examined: China, Yunnan Province, Honghe Autonomous Prefecture, Honghe County, on the roadside (23°16′32.26″ N, 102°25′30.37″ E, altitude 1544.29 m), on dead branches of bamboo in a terrestrial environment, 28 October 2020, H.B. Jiang, HONGHE012 (HMAS 249944, holotype), ex-type living culture, KUMCC 21-0020.

Notes: An ITS nucleotide blast search found the new isolate to be closely related to *Versicolorisporium triseptatum* HHUF 28815 (89.19% similarity), *Neooccultibambusa thailandensis* MFLUCC 16-0274 (88.27% similarity), and *Massarina* sp. MUT 4860 (87.65% similarity), while LSU and TEF1-α nucleotide blast searches indicated that this new isolate belongs to *Occultibambusa*. *Occultibambusa hongheensis* is most similar to *O. maolanensis* but differs in having pale brown ascospores with a broad mucilaginous sheath, longer asci (78–137 μm vs. 66–94 μm) [10], and smaller ascostromata (400–550 μm diam. vs. 544–600 µm diam.) [10]. Based on multi-locus phylogenetic analyses, *O. hongheensis* is sister to *O. maolanensis* with high statistical support (100% ML, 1.00 PP; Figure 1). There are 14 base pair (1.54%; not including gaps) differences between *O. hongheensis* and *O. maolanensis* in comparing a total of 910 nucleotides across the TEF1-α region.

#### 3.2.2. *Occultibambusa kunmingensis* C.X. Liu, H. Zhang & K.D. Hyde in Dong et al., Fungal Diversity 105: 471

**Figure 3:**
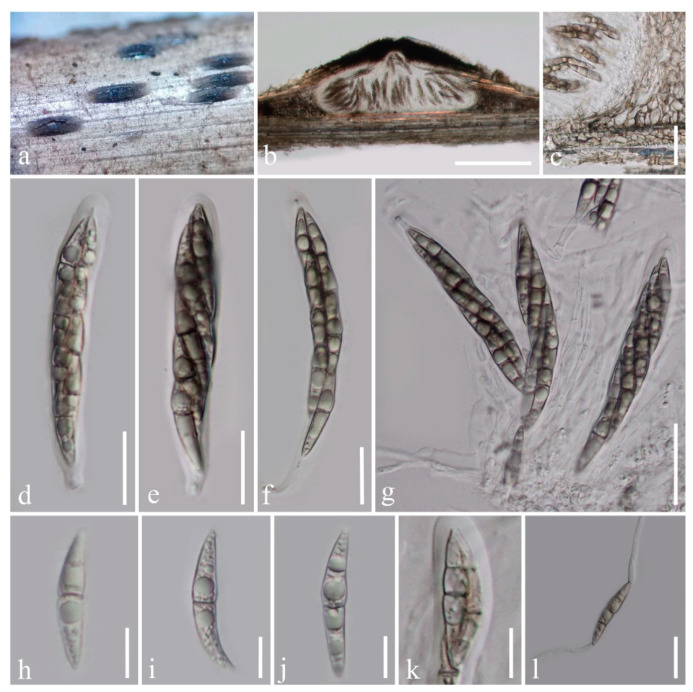
*Occultibambusa kunmingensis* (KUN-HKAS 112011). (**a**) Ascostromata on a dead bamboo branch; (**b**) Vertical section of ascostroma with ostiole; (**c**) Peridium; (**d**–**g**) Asci [(**g**) Asci with pseudoparaphyses]; (**h**–**k**) Ascospores; (**l**) Germinating ascospore. Scale bars: (**b**) = 200 μm; (**c**,**g**) = 30 μm; (**d**–**f**,**l**) = 20 μm; (**h**–**k**) = 10 μm.

Index Fungorum number: IF557930; Facesoffungi number: FoF09272; Figure 3

Holotype: HKAS 102151 

*Saprobic* on dead branches of bamboo, visible as raised, navicular black spots on the host. Sexual morph: *Ascostromata* 170–220 μm high, 350–550 μm diam., solitary, scattered or gregarious (in-group, 2–3 ascomata), immersed under host’s cortex, raised to superficial, ampulliform, flattened at the base, uni-loculate, dark brown to black, coriaceous, with a short, central, minutely papillate ostiole protruding the host. *Peridium* 30–120 μm thick, of unequal thickness, thin at the base, thicker at the sides, composed of several layers of brown pseudoparenchymatous cells, fused with host tissues, arranged in a *textura angularis*, with palisade-like cells at the sides. *Hamathecium* dense, composed of 2.4–3 μm wide, septate, branched, cellular pseudoparaphyses. *Asci* (76–)83–106(–115) × (11–)12–14(–15) μm (*x* = 95 × 13.2 μm, *n* = 20), eight-spored, bitunicate, fissitunicate, cylindric-clavate to clavate, with a short pedicel or subsessile, apically rounded with a narrow, well-developed ocular chamber. *Ascospores* (30–)34–36(–37.5) × (4.5–)5–6 μm (*x* = 35.7 × 5.6 μm, *n* = 20), overlapping 1–2-seriate, or twisted, brown to dark brown, fusiform, with acute ends,1–(3)-septate, occasionally the upper cell larger and longer than the lower cell, straight to slightly curved, with 1–2 large guttules in each cell, lacking a mucilaginous sheath. Asexual morph: Undetermined.

Culture characteristics: Ascospores germinating on PDA within 24 h and germ tubes produced from both ends of ascospore. Colonies growing slowly on PDA, reaching 20 mm in three weeks at room temperature under normal light conditions. Cottony, circular, raised, dark brown from above and below. Mycelium superficial to immersed in media, with branched, septate, smooth hyphae.

Material examined: China, Yunnan Province, Xishuangbanna Dai Autonomous Prefecture, near Bubeng Field Station-Xishuangbanna Station for Tropical Rain Forest Ecosystem Studies, on dead branches of bamboo in the terrestrial environment, 25 January 2019, H.B. Jiang & R. Phookamsak, BN009 (KUN-HKAS 112011; HMAS 249943), living culture, KUMCC 21-0019.

Known host and habitats: bamboo, freshwater, and terrestrial ([13], this study).

Known distribution: Yunnan, China ([13], this study).

Notes: Our collection is morphologically similar to *Occultibambusa kunmingensis*. Based on nucleotide comparisons of ITS, LSU, and SSU pairwise [23], the new isolate has consistent base pairs in comparison to the type strain of *O. kunmingensis*. Thus, we identify the new collection as *O. kunmingensis*. *Occultibambusa kunmingensis* was reported as a saprobe on decaying bamboo submerged in freshwater habitats in Yunnan, China [13] and it has never been reported from terrestrial habitats. Thus, we report this species as a saprobe on bamboo in terrestrial habitat for the first time, suggesting that this species can live in both terrestrial and/or aquatic environments. Alternatively, the freshwater records may have resulted from bamboo recently falling in water.

#### 3.2.3. *Seriascoma bambusae* H.B. Jiang, K.D. Hyde & Phookamsak, sp. nov.

**Figure 4:**
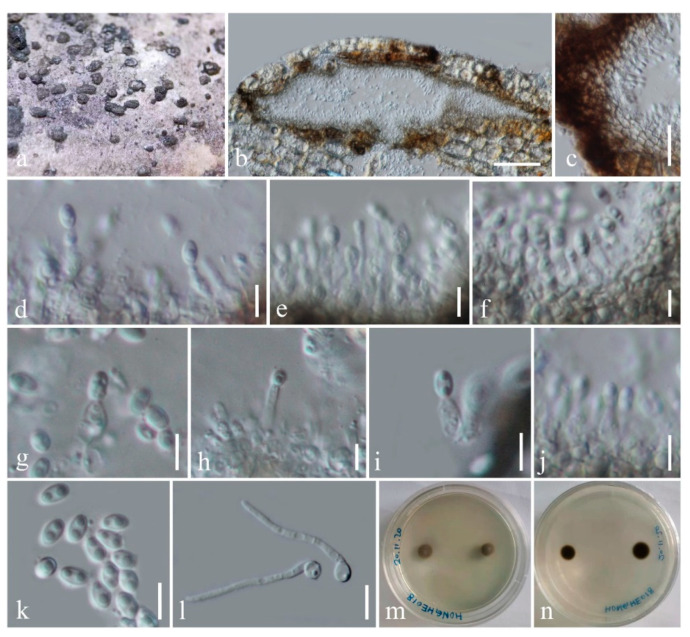
*Seriascoma bambusae* (KUN-HKAS 112014, holotype). (**a**) Conidiomata on surface of dead bamboo culms; (**b**) Vertical section of conidioma; (**c**) Wall of conidioma; (**d**–**j**) Conidiogenous cells bearing conidia; (**k**) Conidia; (**l**) Germinating conidia; (**m**,**n**) Culture from above and reverse. Scale bars: (**b**) = 50 μm; (**c**) = 20 μm; (**l**) = 10 μm; (**d**–**k**) = 5 μm.

Index Fungorum number: IF558430; Facesoffungi number: FoF 09885; Figure 4

Etymology: The specific epithet “*bambusae*” refers to the host, bamboo, on which the new species was collected.

Holotype: KUN-HKAS 112014

*Saprobic* on dead culms of bamboo. Sexual morph: Undetermined. Asexual morph: Coelomycetous. *Conidiomata* 170–380 μm diam., 110–150 μm high, solitary to gregarious, immersed under the host’s cortex, raised, becoming superficial, dull, black, elongate-conical to lenticular or dome-shaped, uni- to bi-loculate, glabrous. *Locules* 95–220 μm diam., 35–140 μm high, clustered, dark brown to black, subglobose. *Peridium* 10–35 μm thick, thin- to thick-walled, of unequal thickness, thick at the sides, thin at the base, composed of host and fungal tissue, with several layers of dark brown to black, pseudoparenchymatous cells of *textura angularis*. *Conidiophores* reduced to conidiogenous cells. *Conidiogenous cells* 5.6–7.2 × 1.6–3.5 μm (*x* = 6.4 × 2.5 μm, *n* = 20), enteroblastic, phialidic, determinate, discrete, cylindrical to ampulliform, hyaline, aseptate, smooth-walled. *Conidia* 3.5–4 × 2–2.3 μm (*x* = 3.8 × 2.2 μm, *n* = 20), subglobose to ellipsoidal, hyaline, 2-guttulate, aseptate, smooth-walled.

Culture characteristics: Conidia germinating on PDA within 24 h. Colonies were growing slowly on PDA, reaching 5 mm in one week at room temperature (10–20 °C), under normal light conditions, colonies cottony, circular, raised, greyish to dark brown from above and below. Mycelium superficial or immersed in media, with branched, septate, smooth hyphae.

Material examined: China, Yunnan Province, Honghe Autonomous Prefecture, Honghe County, on roadside (23°11′40.61″ N, 102°23′6.73″ E, altitude 2012.36 m), on dead culms of bamboo in terrestrial environment, 28 October 2020, H.B. Jiang, HONGHE018 (KUN-HKAS 112014, holotype) *Ibid*. (HMAS 249945, isotype), ex-type living culture, KUMCC 21-0021.

Notes: *Seriascoma bambusae* is typical of the asexual morph of *Seriascoma* in having immersed, eustromatic conidiomata and enteroblastic, phialidic, cylindrical to ampulliform, hyaline, aseptate conidiogenous cells bearing hyaline conidia. *Seriascoma bambusae* is most similar to *Seriascoma* sp. (KUMCC 21-0007) in having multi-loculate conidiomata [38], while *S. didymosporum* has uni-loculate conidiomata. However, *S. bambusae* can be distinguished from *Seriascoma* sp. (KUMCC 21-0007) in having smaller conidiomata (170–380 μm diam. vs. 320–510 μm diam.) and smaller, subglobose conidia (3.5–4 × 2–2.3 μm vs. 4.5–5 × 2–2.4 μm) [38]. Pairwise nucleotide comparison of ITS and TEF1-α sequence data also showed that *S. bambusae* differs from *Seriascoma* sp. (KUMCC 21-0007) in 22/ 502 bp (4.38%) and 26/ 928 bp (2.80%), respectively.

## 4. Discussion

Species of *Occultibambusa* have been discovered in both freshwater and terrestrial habitats (Table 2). Presently, all *Occultibambusa* species have been reported as saprobes on dead bamboo, indicating that the host preference of the genus is restricted to bamboo. *Occultibambusa* has currently been reported from China and Thailand (Table 2). More than 1500 bamboo species are distributed worldwide [39], especially in subtropical and tropical regions [40] Therefore, there is a high potential to discover more new species of the genus from bamboos in other regions [41]. Most species in *Occultibambusa* have similar morphology, but they can be distinguished by dimensions of ascostromata, asci and ascospores and color of ascospores (Table 2). In addition, significant phylogenetic distances of ITS, TEF1-α, and RPB2 can also be used.

The phylogenetic placement of *Occultibambusa fusispora* is unstable in several previous publications. *Occultibambusa fusispora* was separated from all *Occultibambusa* species and *Versicolorisporium triseptatum* in Dong et al. [13] and Wanasinghe et al. [15], while Phukhamsakda et al. [7] showed that *Occultibambusa fusispora* clustered with *O. maolanensis* and *Versicolorisporium triseptatum*. Therefore, in order to resolve this issue, we re-sequenced the ex-type living culture of *Occultibambusa fusispora*. Previously, Dai et al. [4] did not sequence the SSU region of this species, while we sequenced SSU, ITS, LSU, TEF1-α, and RPB2 regions. In our phylogeny, the newly generated sequences of *O. fusispora* (MFLUCC 11-0127II) are consistent with MFLUCC 11-0127 (100% ML, 1.00 PP; Figure 1) and separated well from all *Occultibambusa* species and *Versicolorisporium triseptatum* with high statistical support (98% ML, 1.00 PP; Figure 1).

*Occultibambusa fusispora* matches the typical morphology of sexual morph of *Occultibambusa*; however, it cannot be compared with asexual morphs of other *Occultibambusa* species because *O*. *fusispora* is the only species of this genus known in its holomorph, as the asexual morph was induced on bamboo pieces *in vitro*. In addition, our phylogeny showed *O. fusispora* is basal to *Occultibambusa* and *Versicolorisporium* clade. Therefore, in order to give a more reliable explanation for the placement of *Occultibambusa fusispora*, further studies on *Occultibambusa* species had better be focused on the induction of asexual morph sporulation *in vitro*. Induction of asexual morph sporulation *in vitro* can be performed by following the method described in Phookamsak et al. [42].

In the present study, *Occultibambusa maolanensis* and *O. hongheensis* clustered with *Versicolorisporium triseptatum* and were separated from the main *Occultibambusa* clade with low statistical support (73% ML, 0.71 PP; Figure 1). In addition, the nucleotide BLAST search of SSU sequence of *V. triseptatum* indicated that *V. triseptatum* has consistent base pairs with *O. maolanensis*. The phylogenetic position of *O. maolanensis* and *V. triseptatum* concurs with the studies of Dong et al. [13] and Wanasinghe et al. [15]. *Occultibambusa maolanensis* and *O. hongheensis* cannot be compared with *Versicolorisporium triseptatum* as they are known from different morphs. *Occultibambusa maolanensis* and *O. hongheensis* have the typical morphology of the sexual morph of *Occultibambusa*. The asexual morph of *Occultibambusa* is very different from *Versicolorisporium*. Therefore, the congeneric status of *Occultibambusa* and *Versicolorisporium* is pending further studies.

*Versicolorisporium* is a poorly known coelomycetous genus with *V. triseptatum* collected in Japan on dead culms of *Pleioblastus chino* and *Sasamorpha borealis* (bamboo) [14]. Fresh collections and sequencing of *Versicolorisporium* are needed in order to solve its confusing phylogenetic placement.

*Serisacoma* is presently known as saprobic on bamboo and dead and decaying wood in the terrestrial or freshwater habitats distributed in China and Thailand [4,12,13,38]. The genus accommodates only three species, suggesting that more taxa await discovery [41]. The sexual morphs of *Seriascoma* can be distinguished based on dimensions of ascostromata and ascospores, and the number of locules. The asexual morphs of *Seriascoma* can be distinguished based on dimensions of conidiomata and conidia, the number of locules, and the shape of conidia (Table 3).

## Figures and Tables

**Table 2:** Synopsis of morphological characteristics of *Occultibambusa*.

Species Name	Sexual Morph			Origin	Host	Habitat	References
	Ascostromata	Asci	Ascospores				
*Occultibambusa aquatica*	180–280 × 100–250 μm, subglobose, brown to dark brown, papillate ostiole	73–86 × 9–13 μm, clavate, with a short furcate pedicel	19–25 × 3.5–6.5 μm, 2-seriate, narrow fusiform with acute ends, 1-septate, not constricted at the septum, brownish, with sheath	Chiang Rai, Thailand	Submerged bamboo	Freshwater	[8]
*O. bambusae*	400–550 × 150–200 μm, subglobose, dark brown to black, papillate ostiole	(50–)60–80(−90) × (9.5–)11.5–14.5(−15) μm, broadly cylindrical, with a short furcate pedicel	(22–)23.5–27.5 × 4.5–7 μm, 2–3-seriate, slightly broad fusiform, 1-septate, not constricted at the septum, dark brown, with sheath	Chiang Rai, Thailand	Dead bamboo	Terrestrial	[4]
*O. chiangraiensis*	352–520 × 195–295 μm, depressed globose to subglobose, brown to light brown, ostiole with a slit-like opening	47–92 × 12–16 μm, clavate-oblong, with a short pedicel	16–24 × 5–7 μm, 2-seriate, pale brown to reddish brown, fusiform, tapering towards the ends, (1–)3-septate, not constricted at the septa, without any mucilaginous sheaths and appendages	Chiang Rai, Thailand	Dead stem of *Bambusoideae* sp.	Terrestrial	[8]
*O. fusispora*	240–275 × 135–185 μm, conical with wedged sides, brown to dark brown, papillate ostiole	(60–)65–90(−110) × (11–)12–14(−15)(−16) μm, clavate to cylindric-clavate, with a short furcate pedicel	(20–)22–25(−26) × 5–6(−6.5) μm, 2-seriate, fusiform with acute ends, light brown, 1–(2–3)-septate, not constricted at the septa, without any mucilaginous sheaths and appendages	Chiang Rai, Thailand	Dead bamboo	Terrestrial	[4]
*O. hongheensis*	400–550 × 180–340 µm, ampulliform, conical to subglobose, black, ostiolate	(78–)80–130(–137) × (18–)19–23(–25) μm, cylindrical to clavate, with a short pedicel	(25–)27–30 × (5.5–)8–9(–10) μm, 2-seriate, inequilateral-fusiform, pale brown, 1-septate, slightly constricted at the septum, with a broad mucilaginous sheath	Yunnan, China	Dead bamboo	Terrestrial	This study
*O. jonesii*	200–260 × 196–236 µm, subglobose, dark brown, papillate ostiole	(65–)75–89(–105) × 13.5–19 µm, broadly cylindrical to clavate, with a short pedicel	27–33.5 × 5.5–6.5 µm, 1–3-seriate, inequilateral-fusiform, brown to grayish, 1-septate, constricted at the septum, without any mucilaginous sheaths and appendages	Guizhou, China	Dead bamboo	Terrestrial	[10]
*O. kunmingensis*	220–260 × 110–150 μm, ellipsoidal, black, ostiolate	110–140(–160) × 13–16.5 μm, cylindric-clavate, with a short to long pedicel	32–40 × 5–6.5 μm, 3–4-seriate, fusiform, brown, 1-septate, constricted at the septum, without any mucilaginous sheaths and appendages	Yunnan, China	Submerged bamboo	Freshwater	[13]
	350–550 × 170–220 μm, ampulliform, dark brown to black, minutely papillate ostiole	(76.4–)83–106(–115) × (11–)12–14(–15) μm, cylindric-clavate to clavate, with a short pedicel or subsessile	(30–)34–36(–37.5) ×(4.5–)5–6 μm, 1–2-seriate, fusiform, brown to dark brown, 1–(3)-septate, slightly constricted at the septum, lacking a gelatinous sheath	Yunnan, China	Dead bamboo	Terrestrial	This study
*O. maolanensis*	544–600 µm diam., subglobose to slightly conical, dark brown, papillate ostiole	(66–)77–85(–94) × 17–20(–24) µm, broadly cylindrical to clavate, with a short pedicel	25–31 × 8–10 µm, 2–4-seriate, inequilateral-fusiform, light brown, 1-septate, slightly constricted at the septum, without any mucilaginous sheaths and appendages	Guizhou, China	Dead bamboo	Terrestrial	[10]
*O. pustula*	200–300 ×150–200 μm, conical, black, ostiolate	80–105 × 8–12 μm, cylindrical, with a short furcate pedicel	22–25 × 5–5.5 μm, 2–3-seriate, slightly broad-fusiform, hyaline to pale brown, 1-septate, not constricted at the septum, with sheath	Chiang Rai, Thailand	Dead bamboo	Terrestrial	[4]
	320–350 ×190–220 μm, ellipsoidal, black, papillate ostiole	(60–)78–125 × 12.5–15.5 μm, mostly broadly clavate or sometimes narrowly clavate, with a short or long pedicel	22–29 × 6–8 μm, 1–2-seriate, fusiform, pale brown and 1-septate when young, dark brown and 3-septate when mature, constricted at the septa, without sheath	Yunnan, China	Submerged wood	Freshwater	[13]

**Table 3:** Synopsis of morphological characteristics of *Seriascoma*.

Species Name	Sexual Morph			Asexual Morph			References
	Ascostromata	Asci	Ascospores	Conidiomata	Conidiogenous Cells	Conidia	
*Seriascoma bambusae*	N/A	N/A	N/A	170–380 μm diam., 110–150 μm high, uni- to bi-loculate	5.6–7.2 × 1.6–3.5 μm	3.5–4 × 2–2.3 μm, subglobose to ellipsoidal	This study
*S. didymosporum*	1000–1900 μm diam., 150–320 μm high, multi-loculate	(56–)60–75(−80) × 8–11(−13) μm	11–12(−14.5) × 3–4 μm, clavate to fusiform, with upper cell shorter and wider than lower cell	250–470 μm diam., 110–170 μm high, uni-loculate	4–7(−8) × 1.5–3 μm	4–5.5 × 1.5–2 μm, oblong, with rounded to obtuse ends	[4]
	200–250 μm diam., 120–170 μm high, uni-loculate	70–95 × 9–11 μm	10.5–14.5 × 3.5–5 μm, clavate to fusiform, with upper cell shorter and wider than lower cell	N/A	N/A	N/A	[13]
*S. yunnanense*	275–400 μm diam., 175–205 μm high, uni-loculate	44–83 × 10–20 μm	22–30 × 5–7.2 μm, slightly broad and fusiform, with upper cell larger than lower cell, surrounded by a gelatinous sheath	N/A	N/A	N/A	[12]

## Data Availability

All sequences generated in this study are deposited in GenBank (Table 1). The finalized alignment and tree were submitted to TreeBASE (submission ID: 28553, https://www.treebase.org/ (accessed on 20 July 2021)). Specimens of new taxa and new collections obtained for this study have been deposited in the herbarium of Cryptogams Kunming Institute of Botany Academia Sinica (KUN-HKAS), Yunnan, China and the Herbarium Mycologicum Academiae Sinicae (HMAS), Beijing, China. Living cultures have been deposited in the China General Microbiological Culture Collection Center, Beijing, China (CGMCC) and Kunming Institute of Botany Culture Collection, Kunming, China (KUMCC).
